# Rainfall Is a Risk Factor for Sporadic Cases of *Legionella pneumophila* Pneumonia

**DOI:** 10.1371/journal.pone.0061036

**Published:** 2013-04-16

**Authors:** Carolina Garcia-Vidal, Maria Labori, Diego Viasus, Antonella Simonetti, Dolors Garcia-Somoza, Jordi Dorca, Francesc Gudiol, Jordi Carratalà

**Affiliations:** 1 Department of Infectious Diseases, Hospital Universitari de Bellvitge, IDIBELL (Institut Dinnvestigació Biomèdica de Bellvitge), Universitat de Barcelona, Barcelona, Spain; 2 REIPI (Spanish Network for Research in Infectious Diseases), Madrid, Spain; 3 Department of Microbiology, Hospital Universitari de Bellvitge, Barcelona, Spain; 4 Department of Respiratory Medicine, Hospital Universitari de Bellvitge, Barcelona, Spain; University of Louisville, United States of America

## Abstract

It is not known whether rainfall increases the risk of sporadic cases of *Legionella* pneumonia. We sought to test this hypothesis in a prospective observational cohort study of non-immunosuppressed adults hospitalized for community-acquired pneumonia (1995–2011). Cases with *Legionella* pneumonia were compared with those with non-*Legionella* pneumonia. Using daily rainfall data obtained from the regional meteorological service we examined patterns of rainfall over the days prior to admission in each study group. Of 4168 patients, 231 (5.5%) had *Legionella* pneumonia. The diagnosis was based on one or more of the following: sputum (41 cases), antigenuria (206) and serology (98). Daily rainfall average was 0.556 liters/m^2^ in the *Legionella* pneumonia group vs. 0.328 liters/m^2^ for non-*Legionella* pneumonia cases (p = 0.04). A ROC curve was plotted to compare the incidence of *Legionella* pneumonia and the weighted median rainfall. The cut-off point was 0.42 (AUC 0.54). Patients who were admitted to hospital with a prior weighted median rainfall higher than 0.42 were more likely to have *Legionella* pneumonia (OR 1.35; 95% CI 1.02–1.78; p = .03). Spearman Rho correlations revealed a relationship between *Legionella* pneumonia and rainfall average during each two-week reporting period (0.14; p = 0.003). No relationship was found between rainfall average and non-*Legionella* pneumonia cases (−0.06; p = 0.24). As a conclusion, rainfall is a significant risk factor for sporadic *Legionella* pneumonia. Physicians should carefully consider *Legionella* pneumonia when selecting diagnostic tests and antimicrobial therapy for patients presenting with CAP after periods of rainfall.

## Introduction

*Legionella pneumophila* has been increasingly recognized as a significant cause of sporadic and epidemic community-acquired pneumonia (CAP) in all age groups and in both healthy and immunosuppressed hosts [1]–[6]. *Legionella* pneumonia (LP) is particularly frequent among patients who require admission to an intensive care unit (ICU) [7], [8]. Moreover, *Legionella pneumophila* is the pathogen most frequently associated with early failure caused by inappropriate empirical therapy for pneumonia [9]. In view of its high prevalence, as well as the high morbidity and mortality if treated improperly [10], efforts to achieve early diagnosis and treatment and the prevention of sporadic cases of LP remain a focus of major interest.

Most of what we know about *Legionella* pneumophila transmission has been learned from the investigation of outbreaks [11]–[13], although the majority of cases of LP occur sporadically. Tobacco use, alcohol abuse, chronic lung disease and immunosuppression have been consistently implicated as host risk factors for legionellosis [1], [2]. However, the environmental factors related to sporadic infection have been much more difficult to identify. LP has been related with summertime, especially when humid weather is present [14]. Some researchers have hypothesized that rainfall could be associated with a higher incidence of LP [15], although data supporting this hypothesis are scarce. Moreover, the available information comes from passive surveillance systems and mixes sporadic and epidemic cases. Significantly, no studies have evaluated the impact of rainfall on the incidence of LP compared with the incidence of pneumonia caused by other microorganisms.

We aimed to determine whether sporadic cases of *Legionella* pneumonia are more frequent after rainfall, whereas non-*Legionella* pneumonia is not related with this climatic condition. To this end we analyzed a large prospective cohort of hospitalized patients with CAP.

## Methods

### Setting, Patients, Clinical Assessment and Study Design

This observational study was conducted at an 800-bed tertiary teaching hospital for adults in Barcelona, Spain. The hospital serves an urban area of 900,000 inhabitants. Non-immunosuppressed patients admitted to the hospital with CAP through the emergency department from February 13, 1995 to December 31, 2011 were prospectively recruited and followed up. Patients with neutropenia, solid organ transplantation, chemotherapy, acquired immunodeficiency syndrome or current corticosteroid therapy (≥20 mg prednisone/day or equivalent) at admission were excluded. This observational study was approved by the Institutional Review Board Comité Ético de Investigación Clínica del Hospital Universitari de Bellvitge (Ethics Committee of Clinical Research-Hospital Universitari de Bellvitge) and patients provide their written informed consent to participate in this study.

Patients admitted for community-acquired pneumonia were seen daily during their hospital stay by one or more of the investigators, who recorded clinical data in a computer-assisted protocol. Data were collected on epidemiology, demographic characteristics, comorbidities, causative organisms, antibiotic susceptibilities, biochemical analysis, empirical antibiotic therapy, and outcomes, including mortality. A long-term follow-up visit took place one month after discharge. Pneumonia was defined as an acute illness associated with one or more of the following signs and symptoms: new cough with or without sputum production, pleuritic chest pain, dyspnea, fever or hypothermia, altered breath sounds on auscultation, and leukocytosis, plus the presence of a new infiltrate on a chest radiograph.

For the purposes of the study, patients were divided into two groups: those with pneumonia caused by *Legionella* and those with non-*Legionella* pneumonia. These groups are referred to as the LP group and non-LP group, respectively. Sporadic community-acquired LP was diagnosed with the use of one or more of the following methods: urine antigen test, isolation of *Legionella* in sputum, transthoracic needle aspiration specimen, or pleural fluid, and/or a 4-fold increase in the antibody titer in serologic methods [16]. Cases of community-acquired LP defined as travel-associated in accordance with the criteria of the European Legionnaires’ Disease Surveillance Network were excluded.

### Rainfall Data

Complete precipitation data were obtained from the regional meteorological service (Servei Meteorològic de Catalunya) and were based on continuous rainfall measurements taken at the weather station in our urban area. Total daily rainfall, measured in liters/m^2^, was calculated for the study period and we examined patterns of rainfall over the days prior to admission in each study group. Comparisons were based on two measures: (1) weighted median rainfall (in liters of rain per day/m^2^) over the 2 to 10 days prior to admission; and (2) weighted median rainfall over a two-week period.

### Microbiological Studies and Etiological Diagnosis

Pathogens in blood, normally sterile fluids, sputum, and other samples were investigated using standard microbiological procedures [17]. Isolation of *Legionella* species was attempted in sputum samples and other samples by means of the selective medium buffered charcoal yeast extract-α [18]. *Legionella pneumophila* serogroup 1 antigen in urine was detected by an immunochromatographic method (NOW Legionella Urinary Antigen Test; Binax Inc) or enzyme-linked immunosorbent assay (ELISA-Bartels, Bartels, Trinity Biotech, Wicklow, Ireland) [16], [19]. The *Streptococcus pneumoniae* antigen in urine was detected by using a rapid immunochromatographic assay (NOW Assay; Binax Inc, Portland, Maine). Standard serologic methods were used to determine antibodies against atypical agents. Enzyme immunoassay (EIA) was used to detect antibodies against *L. pneumophila* serogroups 1–6. Microbiological studies were performed at the discretion of physicians.

### Statistical Analysis

Precipitation was assessed with a variable representing the time-weighted rainfall average, taking into account the amount of precipitation over the last 2 to 10 days. This variable was called rainfall average. The range of 2 to 10 days was selected because it corresponds to the incubation period for legionellosis. Rainfall average was calculated using the following weighting criteria: daily precipitation 6 days before admission was assigned a weighting of 20%; daily precipitations 5 and 7 days before were given a weighting of 16%; 4 and 8 days before, 12%; 3 and 9 days before, 8%; and 2 and 10 days before, 4%.

Significant differences between the LP and non-LP groups in relation to rainfall average were detected by means of the Mann-Whitney U test. In addition, a receiver operating characteristic (ROC) curve and the area under the curve (AUC) were used to assess the discriminatory power and predictive value of rainfall average for identifying LP. The relative risks were expressed as odds ratios (OR) and 95% confidence intervals (CI).

Correlations between 15-day rainfall and incidence rates for the LP group and non-LP group were examined using the Spearman correlation coefficient. P values ≤.05 were considered to be statistically significant. Data were analyzed using SPSS (version 15.0; SPSS Inc., Chicago, Illinois) and the R software.

## Results

### General Features of the Study Population

During the 16-year prospective study period, 4393 non-immunosuppressed patients with CAP required hospitalization. Overall, *S. pneumoniae* (1484 cases; 33.8%) was the most frequent causative pathogen, followed by aspiration pneumonia (303 cases; 6.9%) and *Legionella spp.* (231 cases; 5.3%). No *Legionella* epidemic occurred in our hospital area during the study period. We documented 21 cases (9%) as being part of two small clusters of Legionellosis (15 patients in 2002 and 6 patients in 2004). The diagnosis of the 231 included cases of LP was established by using one or more of the following methods: urinary antigen test (206 cases), seroconversion (98 cases), sputum culture (41 cases), transthoracic needle aspiration specimen culture (9 cases), and pleural fluid culture (3 cases).

**Figure 1:**
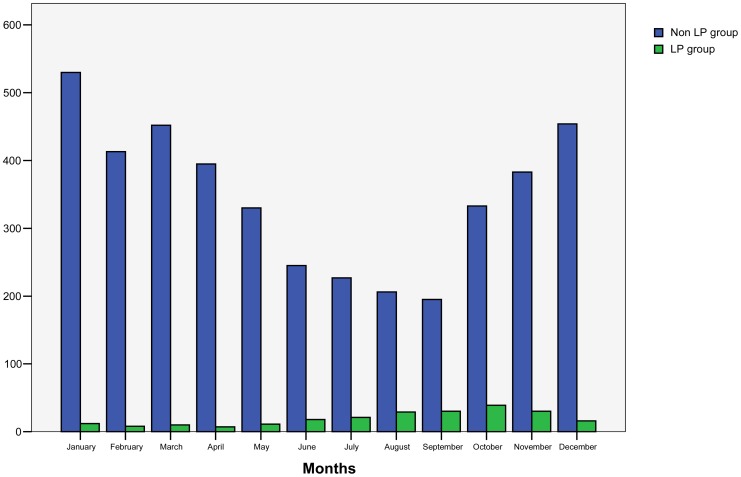
Monthly distribution of LP group and non-LP group patients.

The monthly distribution of LP and non-LP cases is compared in Figure 1. Whereas 72.4% of cases (n = 167) in the LP group occurred during summer and fall (from June to November), only 38.1% of cases in the non-LP group occurred during the same period of time (p<0.001; OR 4.24; 95% CI 3.16–5.69). A clear inverse trend was observed between patients in the LP and non-LP groups.

### Analysis of the Rainfall Effect

**Figure 2:**
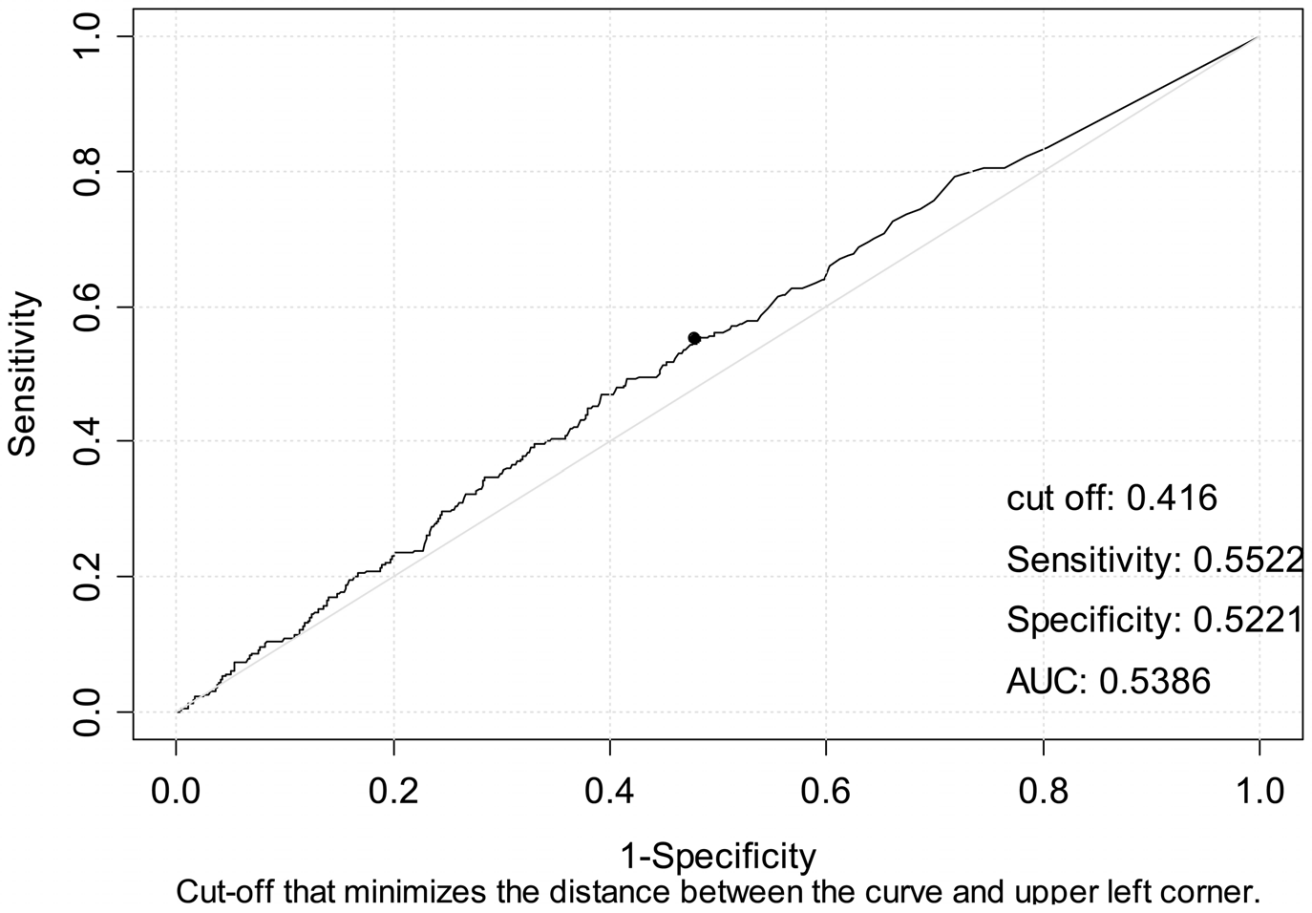
ROC curve of frequency of LP by average rainfall.

Rainfall average in the period that likely preceded infection was associated with the occurrence of LP: median daily rainfall average was 0.556 liters/m^2^ in the LP group versus 0.328 liters/m^2^ in the non-LP group (p = 0.04). Figure 2 shows the ROC curve and AUC used to assess the discriminatory power and predictive value of rainfall average for identifying LP. The likelihood of having LP was higher in those patients admitted on a day with a rainfall average of 0.416 liters/m^2^ (OR 1.349; 95% CI 1.03–1.78; p = 0.03). This correlation was stronger on days with a higher rainfall average.

**Figure 3:**
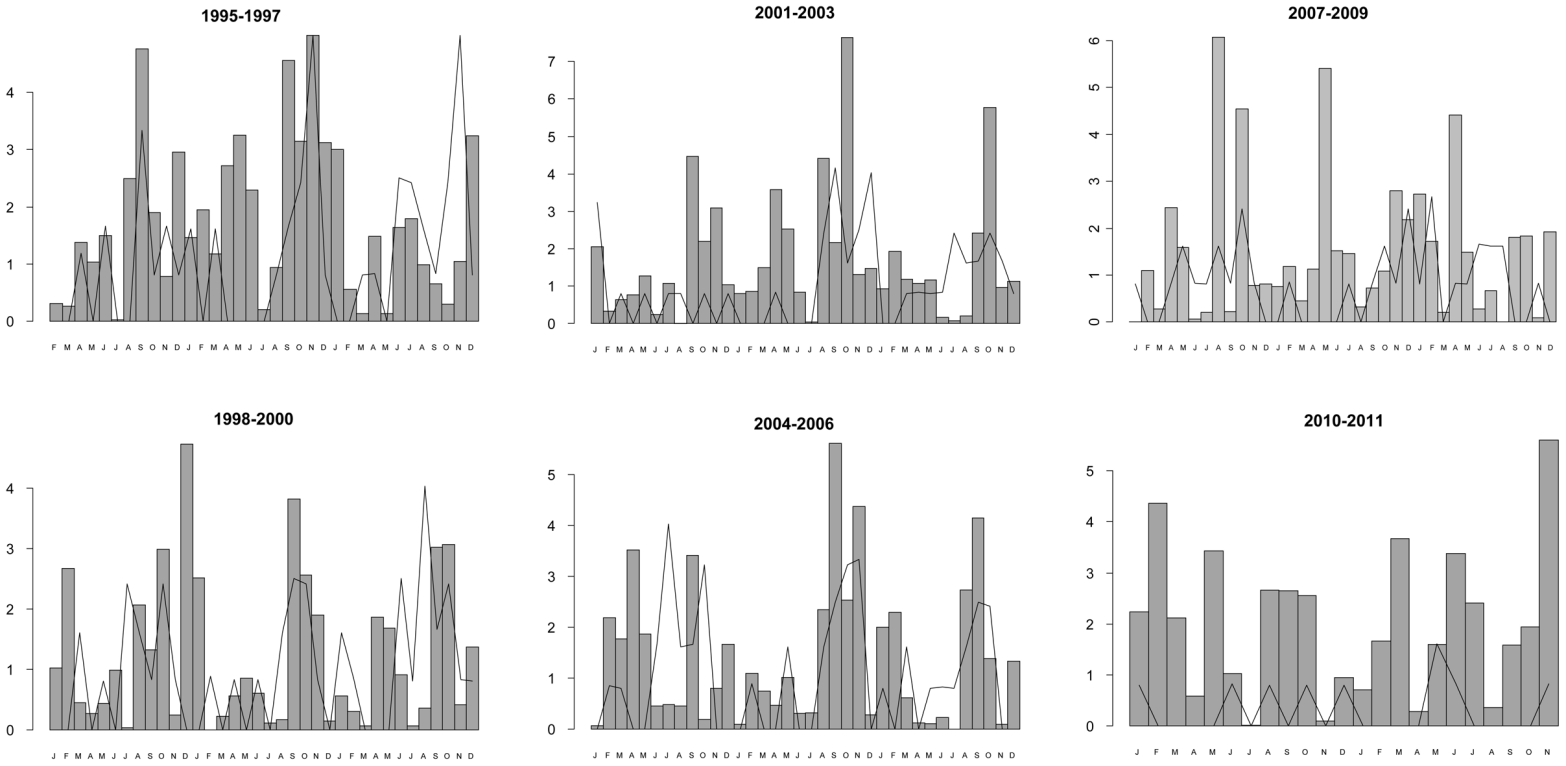
Cases of sporadic LP admitted to our hospital from 1995 through 2011. Cases that occurred during each monthly reporting period are shown in the line graph, while average rainfall is shown in the bar graph.

Figure 3 (a, b, and c) shows the monthly relationship between median rainfall average and patients admitted with LP. Spearman Rho correlations revealed a relationship between cases of LP and rainfall average during each two-week reporting period (0.14; p = 0.003). No relationship was found between cases in the non-LP group and rainfall average (−0.06; p = 0.24).

## Discussion

This prospective study of a large cohort of hospitalized patients with CAP clearly shows that the frequency of LP correlates directly with rainfall and has a distinct seasonal pattern compared with pneumonia caused by other etiologies.

Our findings are consistent with the ecology of *Legionella*, which is a waterborne organism that grows fast in warm temperatures [20], [21]. Although it is unclear exactly how rainfall would lead to increased incidence of LP, an interesting recent study demonstrated that *L. pneumophila* was abundant in transient puddles of rainwater on asphalt roads, especially during warm, rainy weather [22]. Moreover, when there is heavy rain and/or a risk of flooding, each community adopts different measures at its water purification plants in order to protect these structures against the increased turbidity of water, which is caused by dragged sand deposited on the riverbed. Indeed, when faced with such circumstances health authorities in some areas implement measures to change the regime governing the use of water purification plants [23]. In this context, sporadic cases of LP have been linked to potable water supplies [24]. Furthermore, one study reported that having received water from a non-municipal water supply was associated with an increased risk for *Legionella* disease [25].

The timely identification of *Legionella pneumophila* in patients presenting with CAP to the emergency department is important because LP potentially has a high morbidity and mortality rate, which may increase in patients who do not receive adequate antibiotics at admission [9], [10]. However, clinical and laboratory parameters have shown low sensitivity and/or specificity. Although our understanding of CAP caused by *Legionella* has improved substantially in recent years due to new diagnostic and treatment strategies, a recent study conducted by our group [26] showed that 11.2% patients with LP received inappropriate empirical antibiotic therapy at hospital admission. However, the overall proportion of patients who received inappropriate empirical antibiotic therapy at hospital admission remained stable over the years (from 1995 through 2010). Nowadays, the *Legionella pneumophila* urine test is available in most centers, although it may not be available in the emergency department of some institutions 24 hours daily, 7 days per week. Moreover, this test only detects *Legionella pneumophila* serotype 1.

Understanding seasonality of infectious diseases is highly relevant for public health planning [27]. Our findings of a sustained rise in the number of LP cases during warm and rainy periods strongly suggest that strategies for preventing LP need to focus on new environmental sources of *Legionella*. It is, however, unclear which strategies could be beneficial in terms of reducing the incidence of *Legionella* after rainfall. In fact, the feasibility of environmental prevention efforts is questionable, given the ubiquity of rainfall. Nonetheless, it seems reasonable to suggest that plans for assessing the quality of water at water purification plants after severe rainfall should be optimized. Complementarily, it might also be of interest to target the host by increasing empirical antibiotic therapy with *Legionella* coverage during high-incidence periods among patients admitted to the emergency room for pneumonia, especially those possessing other known independent risk factors for LP.

Our study has several limitations that should be acknowledged. The environmental influence on LP incidence is probably multifactorial and we have no information regarding other climatic conditions. Moreover, we focused on CAP patients admitted to our institution, and we cannot rule out the possibility that some patients from our area might have been admitted to other hospitals. Finally, the research was performed at a single institution and only included hospitalized patients. Nevertheless, the study has strengths compared with other research regarding the relationship between climatic conditions and LP [14], [15]. Firstly, all patients were prospective well-documented cases of CAP. This design enabled us to select the true sporadic cases of LP. Secondly, bias due to travel-associated LP was avoided. Thirdly, our study used data on daily rainfall during a long period of time in the area of influence. By using rainfall average we were able to weight the impact of rainfall throughout the incubation period of LP.

To conclude, rainfall is a significant risk factor for sporadic *Legionella* pneumonia. Physicians should carefully consider *Legionella* pneumonia when selecting diagnostic tests and antimicrobial therapy for patients presenting with CAP after periods of rainfall. Our findings provide new insights into the epidemiology of Legionellosis and could help to improve prevention strategies.
